# *Chromobacterium violaceum* in Siblings, Brazil

**DOI:** 10.3201/eid1109.050278

**Published:** 2005-09

**Authors:** Isadora Cristina de Siqueira, Juarez Dias, Hilda Ruf, Eduardo Antonio G. Ramos, Elves Anderson Pires Maciel, Ana Rolim, Laura Jabur, Luciana Vasconcelos, Célia Silvany

**Affiliations:** * Obras Sociais Irmã Dulce, Salvador, Brazil;; † Oswaldo Cruz Foundation, Salvador, Brazil;; ‡ Health Secretariat of the State of Bahia, Salvador, Brazil

**Keywords:** Chromobacterium violaceum, liver abscess, sepsis, Brazil, dispatch

## Abstract

*Chromobacterium violaceum*, a saprophyte bacterium found commonly in soil and water in tropical and subtropical climates, is a rare cause of severe, often fatal, human disease. We report 1 confirmed and 2 suspected cases of *C. violaceum* septicemia, with 2 fatalities, in siblings after recreational exposure in northeastern Brazil.

*Chromobacterium violaceum* is an aerobic, gram-negative bacillus usually found as a saprophyte in soil and water in tropical and subtropical regions (*1*). Despite ubiquitous distribution, human infection with this organism is rare. Since the first human case was described in Malaysia in 1927 (*2*), <150 human cases have been reported worldwide, mainly in Asia, the United States, Australia, and Africa (*3**–**6*). Only 3 cases have been reported in South America, 1 in Argentina (*7*) and 2 in Brazil (*8**,**9*).

Human infection with this organism results in systemic and severe disease with a high fatality rate (*1*). *C. violaceum* infection may begin with cellulitis and skin abscesses (*10**,**11*), with rapid progression to sepsis and multiple organ abscesses, predominantly in lungs, liver, and spleen (*3**–**5*). All previous case reports were of individual, apparently sporadic infections. We report 1 confirmed and 2 suspected cases of systemic *C. violaceum* infection in siblings who shared recreational exposure to stagnant water.

## The Study

In May 2004, 3 cases of sepsis syndrome in children from the same family were reported to the State Health Secretariat of Bahia in northeastern Brazil. The 3 patients had contact with soil and stagnant water in a lake in a rural area of Ilheus municipality, during a day of recreational activity. The 3 brothers spent several hours swimming in the lake with other children and adults, including their parents. Sixty persons were in the group.

Fever, headache, and vomiting developed in patient 1, a previously healthy 14-year-old boy, 2 days after he swam in the lake. He was examined at a local health service; amoxicillin was prescribed and he was sent home. Six days after exposure, he was admitted to a local hospital with fever, dyspnea, and a cervical abscess. The patient's peripheral leukocyte count was 20,000 cells/μL with 5% bands, 78% neutrophils, 14% lymphocytes, 2% eosinophils, and 1% monocytes. Hemoglobin was 11.0 g/dL, aspartate aminotransferase (AST) was 225 U/L, and alanine aminotransferase (ALT) was 120 U/L. Chest radiograph showed diffuse bilateral consolidation, and an abdominal ultrasound showed an enlarged liver. Empiric antimicrobial treatment with oxacillin, ampicillin, and ceftriaxone was initiated. The patient was transferred to the intensive care unit and died of septic shock 36 hours after admission.

**Figure 1:**
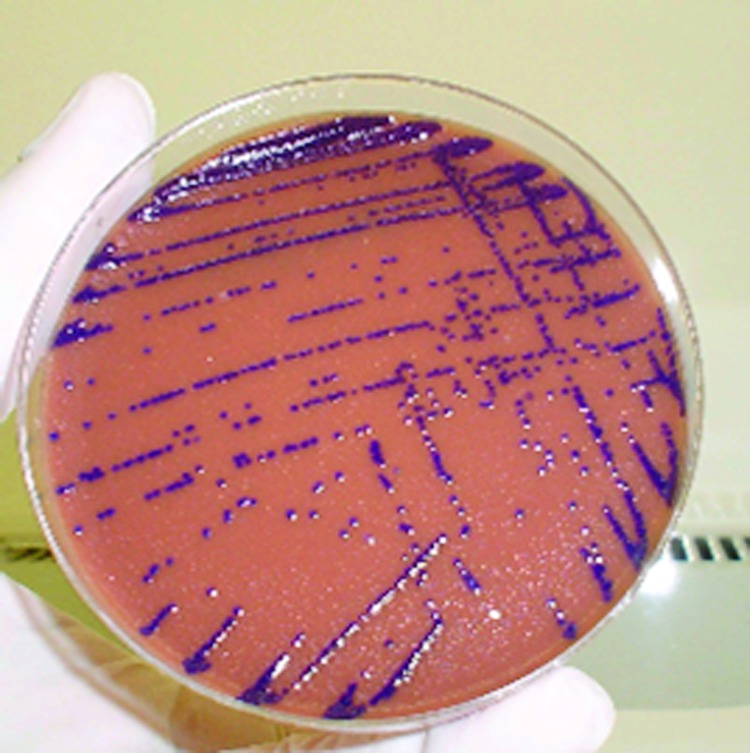
Colonies of *Chromobacterium violaceum* on a chocolate agar plate.

Autopsy showed enlargement of lungs, liver, and spleen with many abscessed areas of suppurative necrosis. An extensive bronchopneumonia was also shown. No spleen lymphoid atrophy was observed. Tracheal aspirate culture yielded smooth purple colonies on chocolate agar (Figure 1), identified as *C. violaceum* by the characteristic dark purple pigment and biochemical profile. Antimicrobial drug susceptibility was determined by disk diffusion. The isolate was resistant to cephalothin, ceftazidime, cefoxitin, and ceftriaxone and was sensitive to trimethoprim-sulfamethoxazole, amikacin, gentamicin, chloramphenicol, ciprofloxacin, and meropenem.

Fever and right earache developed in patient 2, a 12-year-old boy, 3 days after he swam in the lake. He was examined at a local health clinic and sent home. After 2 days, he was admitted to a hospital with purulent discharge in the right ear, fever, facial cellulitis, and diffuse abdominal pain. Leukocyte count was 1,200 cells/μL with 2% bands, 62% neutrophils, 31% lymphocytes, 1% eosinophils, and 4% monocytes. Hemoglobin was 8.0 g/dL, with a platelet count of 158,000 cells/μL. Chest radiograph showed diffuse bilateral consolidation. Empiric treatment with cephalothin and amikacin was initiated, but the patient's condition worsened quickly, and he died 6 hours after admission. No cultures were obtained and autopsy was not performed; therefore, no samples were available for testing. The patient was considered a suspected case-patient on the basis of signs and symptoms and confirmation of the infection in his sibling.

**Figure 2:**
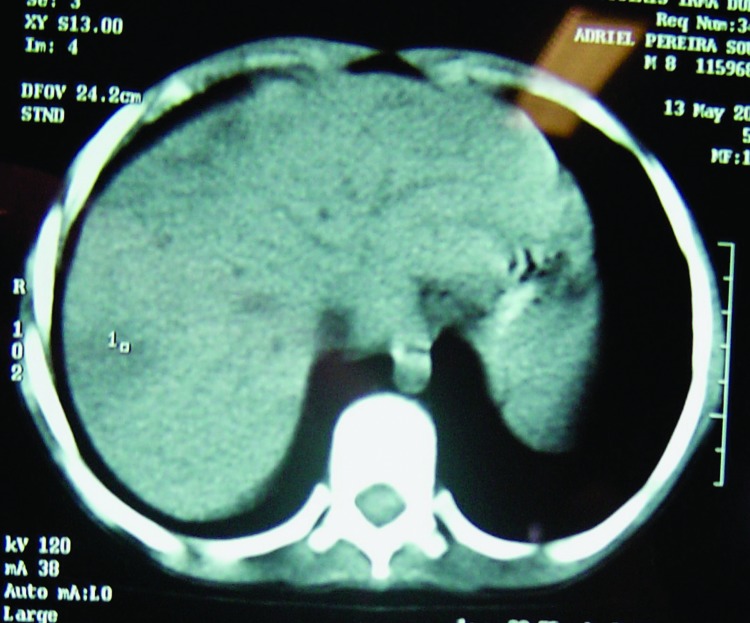
Computed tomographic scan of abdomen of patient 3, showing multiple, small liver abscesses.

Vomiting, abdominal pain, and fever developed in patient 3, a 9-year-old boy, 3 days after he swam in the lake. Like his brothers, he was treated at a local health clinic and admitted to a hospital 3 days afterwards. Leukocyte count was 20,500 cells/μL with 4% bands and 82% neutrophils. Hemoglobin was 11.5 g/dL, AST was 115 U/L, and ALT was 26 U/L. Empiric treatment with ceftriaxone, ampicillin, and metronidazole was initiated. After 48 hours, he was transferred to our institution, the Children's Hospital in Salvador, Bahia. On admission, his abdomen was tender and his liver was enlarged; otherwise, the results of the physical examination were normal. Treatment was changed to ceftazidime, oxacillin, and amikacin. Serial blood cultures were negative for bacteria. A chest radiograph showed perihilar consolidations in both lungs. A computed tomographic scan of the abdomen showed multiple small liver abscesses (Figure 2). Five days after admission, the fever continued in the patient, and cellulitis developed on his left foot and right hand. Antimicrobial therapy was changed to oxacillin plus meropenem. The patient became afebrile after 4 days of meropenem therapy, and symptoms and skin lesions regressed. Studies to rule out underlying immunodeficiency showed no evidence of glucose 6-phosphate dehydrogenase (G6PD) deficiency or HIV infection. The patient received parenteral antimicrobial drug therapy for 6 weeks and an additional 4 weeks of trimethoprim-sulfamethoxazole was prescribed at discharge. He had no symptoms after 3 months of follow-up care and was considered to be a suspected case-patient on the basis of his symptoms and confirmation of the infection in his sibling. Results of the *C. violaceum* culture from case-patient 1 were reported on day 6 of hospitalization.

For microbiologic analysis, samples of water and soil were collected from the lake where the boys had swum. All 6 soil cultures and 4 of 6 water cultures grew *C. violaceum*. Soil and water samples collected near the case-patients' home and neighbors' homes were negative.

## Conclusions

In Brazil, *C. violaceum* is abundant in the water and on the borders of the Negro River in the Amazon basin (*12*); however, this is >1,000 kilometers from the region where the cases occurred. *C. violaceum* infections have been reported at least twice previously in Brazil. In 1984, the organism was cultured from skin abscesses of a young man who had contact with river water in southern Brazil (*8*). In 2000, it was identified from blood culture in a 30-year-old male farm worker who died of severe septicemia associated with multiple lung and liver abscesses (*9*). Most reports worldwide have been associated with rural areas (*5**,**8**,**9*) or stagnant water (*6*).

This report is the first of a cluster of suspected *C. violaceum* infections linked to a common source. Systemic infection caused by *C. violaceum* is rare but severe and is associated with fatality rates >60% (*1**,**13*). Previous reports of *C. violaceum* sepsis have noted fever, hepatic abscesses, and skin lesions, as observed in this cluster. Facial cellulitis and otitis, as observed in patient 2, have also previously been reported (*10*). Only our first case was microbiologically confirmed, but the signs and symptoms and common epidemiologic exposure suggest that all 3 patients had *C. violaceum* infection.

Based on the identification of *C. violaceum* in samples from the lake and onset of symptoms 2–3 days after exposure, we believe that the 3 siblings were exposed while swimming and playing on the banks of the lake. One previous report of 2 cases of *C. violaceum* pneumonia implicated aspiration of fresh water in near-drowning victims (*6*); infection may also have occurred when injured or broken skin is exposed to stagnant water. No cuts or gross abrasions on the skin of the siblings were reported, but microabrasions may have occurred during the recreational activities.

Why these siblings, 3 of 60 persons exposed to the same environment, were the only ones in whom severe illnesses developed is unclear. We hypothesized an underlying factor or familial predisposition to infection. Previously, underlying defects in host defense, especially of neutrophils, have been hypothesized to predispose to infection: cases have been reported in patients with chronic granulomatous disease (*13*) and G6PD deficiency (*14*). However, many case reports describe infections in apparently healthy persons (*5*). The 1 patient tested in this apparent cluster had no detectable immunodeficiency, and his 2 siblings were apparently previously healthy.

Despite their cost, carbapenems may be an appropriate treatment when *C. violaceum* infection is identified. The recommended antimicrobial treatment for *C. violaceum* infection is not well established; some survivors are treated with ciprofloxacin, carbapenems, chloramphenicol with aminoglycoside, or trimethoprim-sulfamethoxazole. When patient 3 was seen in the late stage of infection, meropenem was prescribed empirically for presumptive melioidosis, an infection with *Burkholderia pseudomallei* that may begin similarly to cases in this cluster (*15*). Early recognition and aggressive antimicrobial drug therapy can reduce the high mortality rate associated with both *C. violaceum* infection and melioidosis (*1**,**4**,**15*). Physicians in tropical and subtropical regions should consider *C. violaceum* infection as part of the differential diagnosis of sepsis, especially when associated with skin or multiple organ abscesses or with a history of exposure to stagnant water.

## References

[1] Steinberg JP, Del Rio C. Other gram-negative and gram-variable bacilli. In: Mandell GL, Bennett JE, Dolin R, editors. Principles and practice of infectious diseases, 6th ed. Philadelphia: Churchill Livingstone; 2005. p. 2751–68.

[2] Sneath PH, Whelan JP, Bhagwan SR, Edwards D. Fatal infection by *Chromobacterium violaceum.* Lancet. 1953;265:276–7. 10.1016/S0140-6736(53)91132-5 13085740

[3] Shao PL, Hsueh PR, Hang YC, Lu CY, Lee PY, Lee CH, *Chromobacterium violaceum* infection in children: a case of fatal septicemia with nasopharyngeal abscess and literature review. Pediatr Infect Dis J. 2002;21:707–9. 10.1097/00006454-200207000-00022 12237610

[4] Ti TY, Tan WC, Chong AP, Lee EH. Nonfatal and fatal infections caused by *Chromobacterium violaceum.* Clin Infect Dis. 1993;17:505–7. 10.1093/clinids/17.3.505 8218697

[5] Moore CC, Lane JE, Stephens JL. Successful treatment of an infant with *Chromobacterium violaceum* sepsis. Clin Infect Dis. 2001;32:E107–10. 10.1086/319356 11247733

[6] Ponte R, Jenkins SG. Fatal *Chromobacterium violaceum* infections associated with exposure to stagnant waters. Pediatr Infect Dis J. 1992;11:583–6. 10.1097/00006454-199207000-00013 1528650

[7] Kaufman SC, Ceraso D, Schugurensky A. First case report from Argentina of fatal septicemia caused by *Chromobacterium violaceum.* J Clin Microbiol. 1986;23:956–8. 3711283 10.1128/jcm.23.5.956-958.1986 PMC268759

[8] Petrillo VF, Severo V, Santos MM, Edelweiss EL. Recurrent infection with *Chromobacterium violaceum*: first case report from South America. J Infect. 1984;9:167–9. 10.1016/S0163-4453(84)91234-9 6334119

[9] Martinez R, Velludo MA, Santos VR, Dinamarco PV. *Chromobacterium violaceum* infection in Brazil: a case report. Rev Inst Med Trop Sao Paulo. 2000;42:111–3. 10.1590/S0036-46652000000200008 10810326

[10] Chattopadhyay A, Kumar V, Bhat N, Rao P. *Chromobacterium violaceum* infection: a rare but frequently fatal disease. J Pediatr Surg. 2002;37:108–10. 10.1053/jpsu.2002.29439 11781998

[11] Simo F, Reuman PD, Martinez FJ, Ayoub EM. *Chromobacterium violaceum* as a cause of periorbital cellulitis. Pediatr Infect Dis. 1984;3:561–3. 10.1097/00006454-198411000-00017 6514595

[12] Brazilian National Genome Project Consortium. The complete genome sequence of *Chromobacterium violaceum* reveals remarkable and exploitable bacterial adaptability. Proc Natl Acad Sci U S A. 2003;100:11660–5. 10.1073/pnas.1832124100 14500782 PMC208814

[13] Macher AM, Casale TB, Fauci AS. Chronic granulomatous disease of childhood and *Chromobacterium violaceum* infections in the southeastern United States. Ann Intern Med. 1982;97:51–5. 7092006 10.7326/0003-4819-97-1-51

[14] Mamlok RJ, Mamlok V, Mills GC, Daeschner CW, Schmalstieg FC, Anderson DC. Glucose-6-phosphate dehydrogenase deficiency, neutrophil dysfunction and *Chromobacterium violaceum* sepsis. J Pediatr. 1987;111:852–4. 10.1016/S0022-3476(87)80203-2 3681551

[15] White NJ. Melioidosis. Lancet. 2003;361:1715–22. 10.1016/S0140-6736(03)13374-0 12767750

